# Radiative flow of viscous nano-fluid over permeable stretched swirling disk with generalized slip

**DOI:** 10.1038/s41598-022-15159-w

**Published:** 2022-06-30

**Authors:** Mazhar Hussain, Mudassar Rasool, Ahmer Mehmood

**Affiliations:** 1 Department of Sciences and Humaities, National University of Computer and Emerging Sciences, Lahore, 54770 Pakistan; 2 Department of Mathematics and Statistics, International Islamic University, Islamabad, 4400 Pakistan

**Keywords:** Engineering, Physics

## Abstract

In present years, the study of nanofluids has emerged as a hot topic among the researchers, because the nanoparticle contained in the fluids significantly enhances the heat transfer properties of the fluids. Particularly, rotating ows are of vital importance due to their wide range of scientific, engineering applications, such as jet engines, pumps and vacuum cleaners, as well as geophysical ows. In this study water based nanofluid over radially stretchable rotating disk in the presence of radiation heat transfer is considered. The surface of the stretchable rotating disk surface allows the impact of continuous suction and admits the generalized slip. The Tiwari and Das model is used to describe the nanouid behavior (Tiwari and Das in Int J Heat Mass Transf 50(9–10):2002–2018, 2007). Three types of nanoparticles: Copper (*Cu*), silver (*Ag*) and titanium dioxide $(TiO_2)$ are taken into account. By choosing an appropriate set of similarity transformations, the boundary layer momentum equations and energy equation are transformed to set of nonlinear ordinary differential equations. The impact of emerging quantities like, nanoparticle concentration $\phi$, suction parameter $w_{\circ }$, slip parameters $\zeta$, critical shear stress parameter $\beta$, and radiation parameter $N_{rd}$, are illustrated through several graphs and tables. The Nusselt number and skin friction coefficient are also calculated to analyze the heat transfer process.

## Introduction

Rotation phenomena, in fluid mechanics, has significance due to its emergence in technology and science. For instance, aerodynamics , machinery, gas turbine, thermal power generation, air cleaning, data storage, crystal growth process, medical devices, rotation of galaxy and whirlwinds etc. First, Karman^1^ proposed self-similar transformations which reduce the governing equations of momentum into ordinary differential equations. Cochran^2^ considered cylindrical coordinates and solved the Navier stokes equation numerically by using similarity transformation derived by Karman. Stewartson^3^ was first who study the steady flow of fluid place between two coaxial disk rotating disks. He observed experimentally and theoretically that when both disks rotated in same direction then fluid swirl but when disk rotated in opposite direction the fluid observed almost at rest. Mellor et al.^4^ analyzed experimentally and theoretically, dual disk setup assuming one disk was rotating and second was stationary. Arora^5^ studied steady state heat transfer between two rotating disks in the presence of Newtonian incompressible fluid by using Karman similarity transformation. Kumar et al.^6^ studied the phenomena of fluid flow confined between one rotating disk and one stationary porous solid disk. Anderson et al.^7^ studied the heat transfer for power law fluids in the presence of rotating disk.

Crane^8^ was first who introduce fluid flow over stretching plate. Later Ming et al.^9^ analyzed numerically heat transfer for the time independent incompressible flow of power law fluid over stretching sheet. Later Wang^10^ proposed solution of equation of momentum for the three-dimensional flow over stretching disk. Fang^11^ extended the work of Karman for the stretchable stationary disk and stretchable rotating disk. Later Fang and Tao^12^ analyzed the stretching phenomena unsteady flow viscous fluid over stretchable rotating disk with deceleration.

Nanofluids possesses importance due to their physical properties and uses in technologies. For instance, nanofluids are used for solar thermal conductors, cooling electronics, automobile radiator, thermal storage, refrigeration, light weight concrete, cancer treatment and diagnosis etc. In early fluid mechanics scientists analyzed the thermal and physical properties of fluids without nanoparticles but Choi and Eastman^13^ was first who gave the concept to add up nanoparticles to enhance the thermal properties of fluid. Wang et al.^14^, Kakac and Pramuanjaroenkij^15^ and many other investigated the physical properties of nanofluids. There are different heat models also devolved for analyzing the convective flows for instance, Tiwari and Das^16^, Daungthongsuk and Wongwises^17^, Wang and Wei^18^, Oztop and Abu-Nada^19^ etc. Bachok et al.^20^ analysed boundary layer flow of nano-fluid over moving surface. Mustafa et al.^21^ gave a study of stagnation point flow toward stretching sheet by using homotopy analysis method. Sheikholeslami et al.^22^ analyzed the heat transfer between two rotating disks where lower plate was a stretchable disk and upper plate was a solid permeable disk. Kasaeian et al.^23^ introduced the model of nanofluid in porous medium. Bachok et al.^24^ investigated the steady incompressible flow and heat of viscous fluid over rotating disk. Ashorynejad et al.^25^ studied heat transfer of nanofluid over stretching cylinder in the presence of magnetic field. They observed the heat transfer for nanoparticles of copper, silver, alumina and titanium. Yin et al.^26^ analysed heat transfer in the presence of stretchable rotating disk and nanofluid. Recently Kumar et al.^27^ investigated dusty flow of nanofluid over stretchable swirling disk in the presence of carbon nanotubes with uniform heat source and sink.

Suction play important role in heat transfer and to increase the speed in aerodynamics. So suction is applied to different structures for cooling. Erickson et al.^28^ analyzed the effects of suction on heat and mass transfer for a moving continuous flat plate. Ackroyd^29^ researched on suction or injection in the presence of steady flow of fluid over rotating disk. Ishak et al.^30^ worked on heat transfer in stretching cylinder in presence of suction phenomena. Recently, Ganesh et al.^31^ presented, incompressible time independent flow of viscous fluid between two porous plates, where lower plate observing injection and upper plate observing suction. Hayat et al.^32^, discussed characteristic of activation energy and porosity in time dependent flow of nanofluid in the presence of stretchable rotating disk.In 2021, Rehman et al.^33^ studied slip effect on flow of Casson nanofluid flow over stretchable permeable surface by implementing bvp4c.

Slip is observed at the surface of body. Slip phenomena has great importance due to their use for drag reduction in hydro and aerodynamics. As we know that any surface cannot to be ideally smooth, consequently, every surface observe slip. Navier introduced Navier slip which used widely to stimulate the fluid flows. Thompson and Troian^34^ used the Navier slip model and develop a generalized model known as generalized slip. Ramzan et al.^35^ recently used Thompson and Troian model^34^ to analyze the flow of nano-fluid.The more recent studies comprising of nanofluids and their applications in different areas of science and engineering can be found^36–42^.

After going through above literature survey, the authors observed that very less attention is given to radiation heat transfer of viscous nanofluid when flow is subjected to stretchable swirling disk admitting generalized slip and continuous suction. .

## Problem formulation

**Figure 1:**
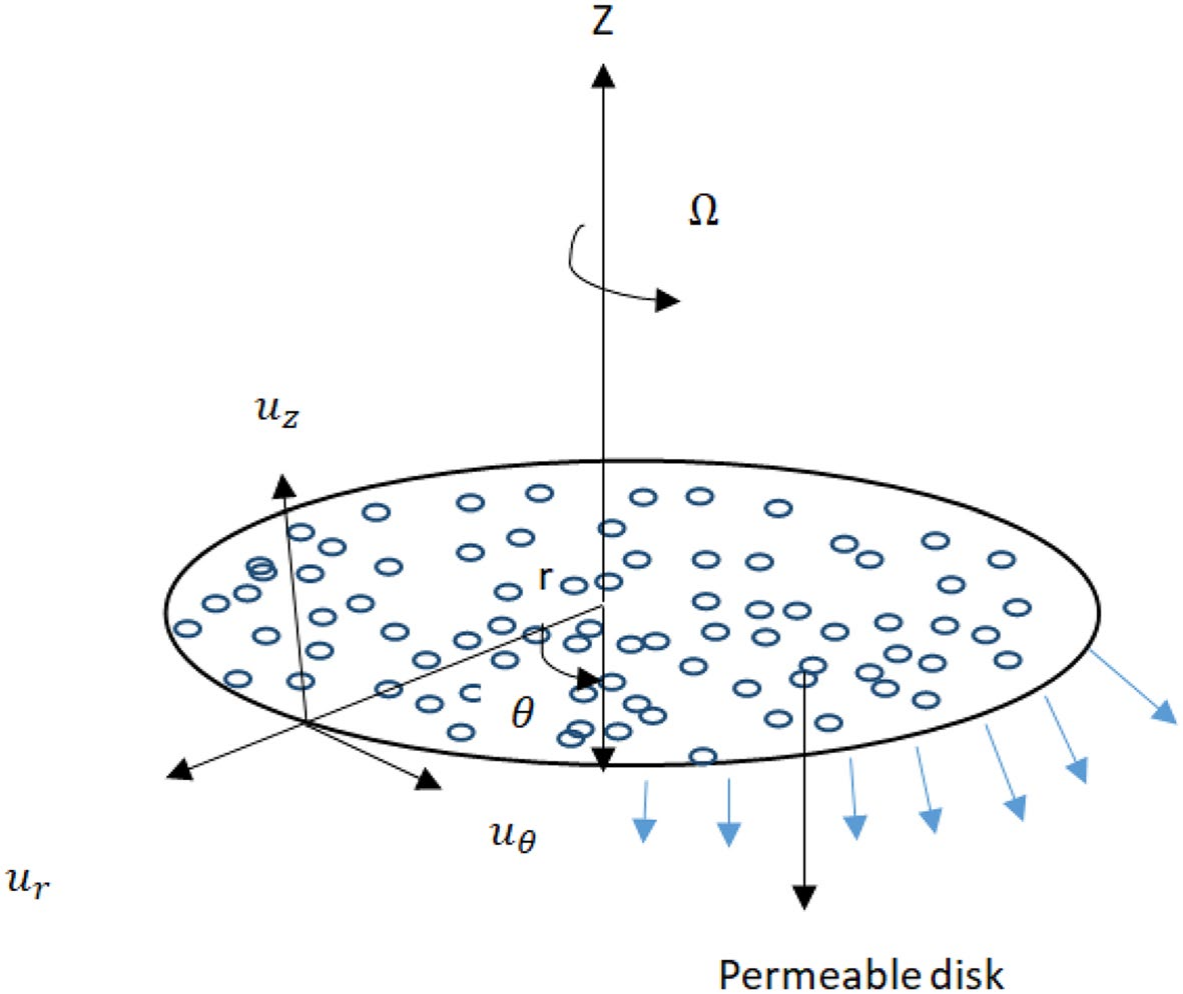
Geometry of flow.

Figure 1 elucidates the time independent, incompressible swirl motion of viscous fluid due to a porous rotating disk admitting slip and radiation. The disk having temperature $T_{w}$ at $z=0$, $T_{\infty }$ is temperature away from disk. Problem is formulated in cylindrical coordinates $(r,\theta ,z)$ in which velocity components $u_r=r\omega , \quad u_\theta =r \Omega$ and $u_z=h_{\circ }$ increase in increasing $r-, \theta - \quad and\quad z-$ axis direction. Where $\omega$ is stretching strength and $\Omega$ is rotation strength. During the modeling of energy equation effects of thermal radiation is assumed. Tiawri Das model is taken into account to investigate heat transfer.

1 $$\begin{aligned}\frac{1}{r} \frac{\partial (ru_r)}{\partial r}+\frac{\partial u_z}{\partial z}=0, \end{aligned}$$

2 $$\begin{aligned}u_r \frac{\partial u_r}{\partial r}+u_z \frac{\partial u_r}{\partial z}-\frac{u_\theta ^2 }{r}=-\frac{1}{\rho _{nf}} \frac{\partial p }{\partial r}+ \nu _{nf}\left( \frac{\partial ^2 u_r}{\partial r^2}+\frac{1}{r}\frac{\partial u_r}{\partial r}+\frac{\partial ^2 u_r}{\partial z^2}-\frac{u_r}{r^2}\right) , \end{aligned}$$

3 $$\begin{aligned}u_r\frac{\partial u_\theta }{\partial r}+u_z\frac{\partial u_\theta }{\partial z} +\frac{u_\theta u_r}{r}=\nu _{nf}\left( \frac{\partial ^2u_\theta }{\partial z^2}+\frac{1}{r}\frac{\partial u_\theta }{\partial r}+\frac{\partial ^2 u_\theta }{\partial z^2}-\frac{u_\theta }{r^2}\right) , \end{aligned}$$

4 $$\begin{aligned}u_r\frac{\partial u_z}{\partial r}+ u_z \frac{\partial u_z}{\partial z}=-\frac{1}{\rho _{nf}}\frac{\partial p}{\partial z}+\nu _{nf} \left( \frac{\partial ^2 u_z}{\partial r^2}+\frac{1}{r}\frac{\partial u_z}{\partial r}+\frac{\partial ^2 u_z}{\partial z^2}\right) , \end{aligned}$$

5 $$\begin{aligned}(\rho _{Cp})_{nf}\left( u_r \frac{\partial T}{\partial r}+u_z\frac{\partial T}{\partial z}\right) =k_{nf}\left( \frac{\partial ^2 T}{\partial r^2}+\frac{1}{r}\frac{\partial T}{\partial r}+\frac{\partial ^2 T}{\partial z^2}\right) -\frac{\partial q_r}{\partial z}, \end{aligned}$$

where $q_{r}=\frac{4\sigma _{1}}{3k_{1} } \frac{\partial T^{4}}{\partial z}=-\frac{16\sigma _{1}}{3k_1}T^3_{\infty }\frac{\partial ^{2}T }{\partial z^2}$ emerges in energy equation due to radiation because $T^4\approx 4T^3_{\infty }T-3T^4_{\infty }$.

Here $\sigma _{1}$ Stefan–Boltzmann constant, $k_{1}$ is absorption coefficient. It is presumed that temperature differences inside the flow adequately meager so that the term $T^{4}$ may be expressed as linear function of temperature. It happened by expanding $T^{4}$ in Taylor series about $T_{\infty }$ and neglecting the second and high order terms.

6 $$\begin{aligned}(\rho _{Cp})_{nf}\left( u_r \frac{\partial T}{\partial r}+u_z\frac{\partial T}{\partial z}\right) =k_{nf}\left( \frac{\partial ^2 T}{\partial r^2}+\frac{1}{r}\frac{\partial T}{\partial r}+\frac{\partial ^2 T}{\partial z^2}\right) + \frac{16\sigma _{1}}{3k_1}T^3_{\infty }\frac{\partial ^{2}T }{\partial z^2} , \end{aligned}$$

comprising of following boundary conditions.

7 $$\begin{aligned}u_r=r \omega +\zeta ^*(1-\beta ^*(r) \tau _w)^{-\frac{1}{2}}\tau _{w},\quad u_{\theta }=r \Omega ,\quad u_{z}=h_{\circ }, \quad T=T_{w} \quad at \quad z=0,\nonumber \\&u_r=0,\quad u_\theta =0,\quad p=p_{\infty }-\omega \mu _{f},\quad T\rightarrow T_{\infty } \quad as \quad z\rightarrow \infty \end{aligned}$$

where $\zeta ^*$ is Navier slip length, $\beta ^*(r)$ is reciprocal of critical shear rate, $\tau _w=\frac{\partial u_r}{\partial z}\big |_{z=0}$ and $h_{\circ }$ is velocity of suction $(h_{\circ }<0)$. The nanofluid properties are defined as given by^16,43^

8 $$ \begin{aligned}    & \mu _{{nf}}  = \frac{{\mu _{f} }}{{(1 - \phi )^{{2.5}} }},\alpha _{{nf}}  = \frac{{k_{{nf}} }}{{(\rho C_{p} )_{{nf}} }}, \\     & \rho _{{nf}}  = (1 - \phi )\rho _{f}  + \phi \rho _{s} , \\     & (\rho C_{p} )_{{nf}}  = (1 - \phi )(\rho C_{p} )_{f}  + \phi (\rho C_{p} )_{s} , \\     & \frac{{k_{{nf}} }}{{k_{f} }} = \frac{{(k_{s}  + 2k_{f} ) - 2\phi (k_{f}  - k_{s} )}}{{(k_{s}  + 2k_{f} ) + \phi (k_{f}  - k_{s} ))}}, \\  \end{aligned}  $$

where ${\mu _{f}}$ represents the dynamic viscosity of fluid, $\rho _{s}$ and $\rho _{s}$ are, respectively, density of base fluid in which nanoparticle are suspended and density of suspended nanoparticle. $(\rho C_{p})_{nf}$ and $(\rho C_{p})_f$ specific heat capacitance of nanofluid and nanoparticle, respectively. $k_{nf}$ expresses effective thermal conductivity of nanofluid.

We are considering the thermophysical properties of base fluid and nanoparticles according to Oztop and Nada model^19^, confined to spherical shaped nanoparticles, are given in Table 1.

**Table 1:** Thermo pysical properties of water and considered nanoparticlas.

Physical properties	$H_2O$	*Cu*	*Ag*	$TiO_2$
Cp/(J/(kg k))	4179	385	235	686.2
$\rho$/(kg/m$^3$)	997.1	8933	10500	4250
*k*/(W/(m k))	0.613	400	429	8.9538

The governing equations (1)–(6) are representing the flow phenomena. The considered problem is self-similar in nature with appropriate choice of critical shear rate $\beta$ . These equations can be transformed to non-linear ordinary differential equations by introducing the following similarity transformations as untilized by Von Karman^1^:

9 $$ \begin{aligned}    & u_{r}  = r\omega F^{\prime}(\eta ),u_{\theta }  = r\Omega G(\eta ),u_{z}  =  - \sqrt {2\omega \nu _{f} } F(\eta ), \\     & p = p_{\infty }  - \omega \mu _{f} P(\eta ),T = T_{\infty }  - (Tw - T_{\infty } )\theta (\eta ),\eta  = z\sqrt {\frac{{2\omega }}{{\nu _{f} }}} . \\  \end{aligned}  $$

In the view of above similarity transformations the continuity equation identically satisfied and governing equations (1)–(6) take their new form as:

10 $$\begin{aligned}\left( \frac{1}{(1-\phi )^{2. 5}(1-\phi )\rho _f+\phi \rho _s/\rho _f}\right) F^{'''}=\frac{1}{2}(F^{'})^{2}-FF^{''}-\frac{1}{2}G^{2}, \end{aligned}$$

11 $$\begin{aligned}\left( \frac{1}{(1-\phi )^{2. 5}(1-\phi )\rho _f+\phi \rho _s/\rho _f}\right) G^{''}=GF^{'}-FG^{'}, \end{aligned}$$

12 $$\begin{aligned}\theta ^{''}+\left( \frac{P_{r}3K_{f}{(1-\phi ) }+\phi (\rho C_{p})_{s}/(\rho _{Cp})_{f} }{k_{nf}(3+4N_{rd})}\right) F\theta ^{'}=0. \end{aligned}$$

with following boundary conditions:

13 $$ F(0) = w_{^\circ } ,\quad F^{\prime}(0) = 1 + \zeta (1 - \beta (r))F^{\prime\prime}(0))^{{ - \left( {\frac{1}{2}} \right)}} F^{\prime\prime}(0),\quad G(0) = c,\quad \theta (0) = 1,\quad F(\infty ) = 0,\quad G(\infty ) = 0,\quad \theta (\infty ) = 0. $$

where ${w_{\circ }}=-\frac{h_{\circ }}{\sqrt{2\omega \nu _{f}}}$ suction parameter$(w_{\circ }>0)$, $N_{rd}=\frac{4 \sigma _{1}}{K_1 K_{nf}} T^3_{\infty }$ is radiation parameter, $\zeta$ and $\beta$ are dimensionless velocity slip and critical shear rate, respectively. which are defined as:

14 $$\begin{aligned} \zeta = \zeta ^*\sqrt{\frac{2 \omega }{\nu _f}},\quad \beta = \beta ^*(r) r\omega \sqrt{\frac{2 \omega }{\nu _f}}, \end{aligned}$$

Following Aziz^44^ results $\zeta$ and $\beta (r)$ must be constant and should not be function of variable *r*. Therefore we consider

15 $$\begin{aligned} \zeta ^*= A\sqrt{\frac{\nu _f}{2 \omega }},\quad \beta ^*(r)=B \sqrt{\frac{\nu _f}{2\omega }}\frac{1}{r\omega }, \end{aligned}$$

here A and B are constants.

The physical quantities of interest are skin friction coefficient and Nusselt number.

The radial wall stress $\tau _{rw}$ and circumferential wall stress $\tau _{\theta w}$ are follows:

16 $$\begin{aligned} \tau _{rw}= & {} \frac{\mu _{f}}{(1-\phi )^{2. 5}} \left( r\omega \sqrt{\frac{2\omega }{\nu _{f}}}\right) F''(0), \end{aligned}$$

and

17 $$\begin{aligned} \tau _{\theta w}= & {} \frac{\mu _{f}}{(1-\phi )^{2. 5}}\left( r\omega \sqrt{\frac{2\omega }{\nu _{f}}}\right) G'(0), \end{aligned}$$

**Skin friction coefficient**

Skin friction coefficient is defined as follows:

18 $$\begin{aligned} C_f=\left( \frac{2\omega r^2}{\nu _f}\right) ^{-\frac{1}{2}} \frac{\sqrt{F''(0)+G'(0)}}{{(1-\phi )^{2. 5}}}. \end{aligned}$$

**Nusselt number**

Nusselt number is defined as follows:

19 $$\begin{aligned} Nu_r=-\sqrt{\frac{2r^2\omega }{\nu _f}}\frac{K_{nf}}{K_f}\theta '(0). \end{aligned}$$

## Method of solution

To solve the equations governing the flow, the MATLAB built in routine namely bvp4c has been utilized. For this, the set of governing equations (10)–(13) are transformed into system of first order ordinary differential equations as described below :

$$\begin{aligned}y(1)=F,\quad y(2)=F',\quad y(3)=F'',\quad y(4)=G,\quad y(5)=G',\quad y(6)=\theta ,\\&\quad y(7)=\theta '. \\&\quad y'(3)=((1-\phi )^{2. 5}(1-\phi )\rho _f+\phi \rho _s/\rho _f))\cdot (0. 5*y(2)^2-y(1)\cdot y(3)-0. 5*y(4)^2), \\&\quad y'(4)=((1-\phi )^{2. 5}(1-\phi )\rho _f+\phi \rho _s/\rho _f))\cdot (y(4)\cdot y(2)-y(5)\cdot y(1)), \\&\quad y'(6)=\left( \frac{k_{nf}(3+4N_{rd})}{P_{r}3K_{f}{(1-\phi ) }+\phi (\rho C_{p})_{s}/(\rho _{Cp})_{f}}\right) \cdot (-y(1)\cdot y(7)). \end{aligned}$$

the boundary conditions are comprised of

$$ \begin{aligned}    & ya(1) - w_{^\circ }  = 0,\quad ya(2) - 1 - \zeta  \cdot ya(3) \cdot (1 - \beta  \cdot ya(3))^{ - } 0.5 = 0, \\     & ya(4) - c\; = 0,\quad ya(6) - 1\; = 0, \\     & yb(2) = 0,\quad yb(4) = 0,\quad yb(6) = 0. \\  \end{aligned}  $$

## Results and discussion

The numerical results of variation of rotation parameter *c*, suction parameter $w_{\circ }$, Navier slip length parameter $\zeta$, parameter of reciprocal of shear rate $\beta$, volume fraction $\phi$ and radiation parameter $N_{rd}$, are disclosed in Tables 2, 3 and 4 and Figs. 2, 3, 4, 5, 6, 7, 8, 9, 10, 11, 12, 13, 14, 15, 16, 17, 18, 19, 20, 21, 22, 23 and 24.

**Table 2:** Numerical analysis of wall stress $\tau _{rw}$, circumferential wall stress and skin friction coefficient $C_{f}$ for different values of $w_{\circ }$, $\zeta$, $\beta$ and *c* with volume fraction $\phi =0.05$ of $TiO_2-water$.

$w_{\circ }$	$\zeta$	$\beta$	c	$\frac{F''(0)}{(1-\phi )^{2. 5}}$	$\frac{G'(0)}{(1-\phi )^{2. 5}}$	$\frac{(F''(0)+G'(0))^{\frac{1}{2}}}{(1-\phi )^{2.5}}$	CPU time
0	0.1	0.2	1	-0.6923	-1.1785	1.3668	141.6250
0.5	0.1	0.2	1	-0.9956	-1.4764	1.7808	147.7031
1	0.1	0.2	1	-1.3474	-1.8431	2.2831	150.8594
1.5	0.1	0.2	1	-1.7248	-2.2706	2.8514	153.2344
2	0	0.2	1	-2.5814	-2.8095	3.8154	156.3750
-	0.6	0.2	1	-1.0791	-2.5814	2.7979	159.1875
-	1.2	0.2	1	-0.6750	-2.5086	2.5978	161.3906
-	1.8	0	1	-0.4744	-2.4700	2.5151	164.0625
-	-	0.8	1	-0.5385	-2.4825	2.5402	166.7813
-	-	1.6	1	2.5402	-2.4948	2.5664	168.9219
-	-	2.4	0	-0.7085	0	0.7085	171.0938
-	-	-	1	-0.6635	-2.5064	2.5928	172.8906
-	-	-	2	-0.5418	-5.1472	5.1757	174.8281
-	-	-	3	-0.3737	-8.0100	8.0187	176.9375

In Table 2, the effects of different values of suction parameter $w_{\circ }$, Navier slip length parameter $\zeta$, reciprocal of critical shear rate $\beta$ and rotation parameter *c* with volume fraction $\phi =0. 05$ of $TiO_2$ on skin friction coefficient are listed. It can be seen, as the magnitude of suction parameter $w_{\circ }$ enhances, the skin friction coefficient also rises and by increasing slip parameter $\zeta$ skin friction coefficient decreases. In opposite fashion, as the critical shear rate coefficient $\beta$ lifts and skin friction start to rise. Similarly, as the rotation parameter *c* mounts, the skin friction intensify.

High magnitude of Nusselt number is noted against $TiO_2$ than *Ag* and *Cu* by changing volume fraction $\phi$, shown in Table 3. Which is evident to high rate of heat transfer for $TiO_2$, further, for the large values of rotation parameter *c* conduction of *Cu* jumps.

**Table 3:** Values of Nusselt number $\frac{k_nf}{K_f}\theta '$ for various values of volume fraction $\phi$ and *c*.

$\phi$	c		$-\frac{k_nf}{K_f}\theta '$			CPU time	
		$TiO_2$	*Ag*	*Cu*	$TiO_2$	*Cu*	*Ag*
0	0.5	1.8515	1.8515	1.8515	125.4375	108.1250	99.2813
0.05	–	1.8772	1.8580	1.8699	154.3281	115.1563	106.3594
0.1	–	1.9038	1.8674	1.8918	158.6094	117.7500	110.8281
0.15	–	1.9327	1.8810	1.9192	162.9375	121.3906	113.8438
0.2	–	1.9655	1.9014	1.9555	169.4531	125.6250	117.2813
0.05	0	1.8670	1.8466	1.8587	176.2813	129.3750	122.8125
–	1	1.9057	1.8895	1.9008	179.7656	131.4688	125.8750
–	2	1.9993	1.9915	2.0014	185.9688	133.6094	128.0625
–	3	2.1176	2.1189	2.1273	189	135.7969	130.4844

For $\zeta =0.1$, $\beta =0.1$, $N_{rd}=0.2$ and , $w_{\circ }=0.1$ and $Pr=6.2$.

Data in Table 4 divulges, as suction parameter escalates the conduction heat transfer become diminish due to thin momentum boundary layer, as $\beta$ progresses the conduction heat transfer get strengthen. As values of $\zeta$ turn up the conduction ascend.

**Table 4:** Effects of suction, navier slip parameter, reciprocal of critical shear stress and radiation on Nusselt number.

$w_{\circ }$	$\zeta$	$\beta$	$N_{rd}$	$-\frac{k_nf}{K_f}\theta '$
0	0.1	0.2	0.3	1.5129
0.5	–	–		3.0581
1	–	–		4.8755
1.5	–	–	–	6.8249
2	–	–	–	8.9007
–	0.3	–	–	8.7637
–	0.6	–	–	8.6988
–	0.9	0	–	8.6556
–	–	0.4	–	8.6669
–	–	0.8	–	8.6774
–	–	1.2	0	12.0660
–	–	–	0.5	7.3327
–	–	–	1	5.2962
–	–	–	1.5	4.1604
–	–	–	2	3.4348

When $\phi =0.05$ and $c=1$ for $TiO_2-water$.

**Figure 2:**
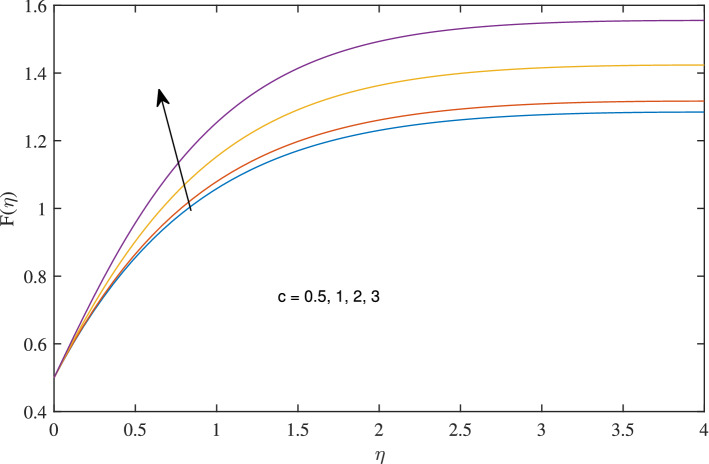
Effects of variation of *c* on axial velocity, when $w_{\circ }=0.5$, $\zeta =0.1$, $\beta =0.2$, $N_{rd}=1$, $\phi =0.1$ and $Pr=6.2$.

**Figure 3:**
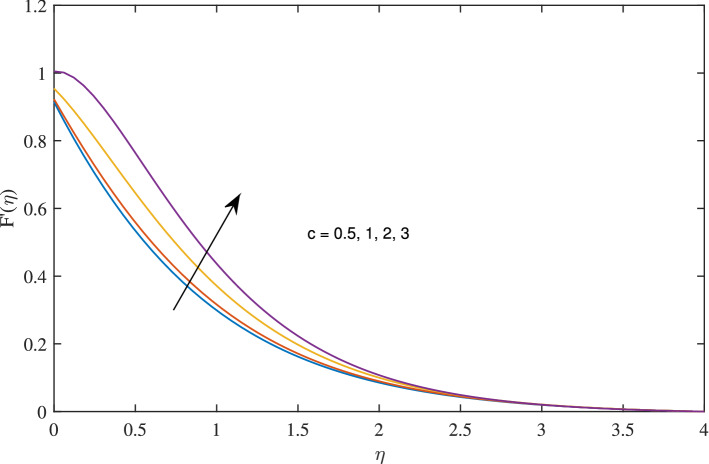
Effects of variation of *c* on radial velocity, when $w_{\circ }=0.5$, $\zeta =0.1$, $\beta =0.2$, $N_{rd}=1$, $\phi =0.1$ and $Pr=6.2$.

**Figure 4:**
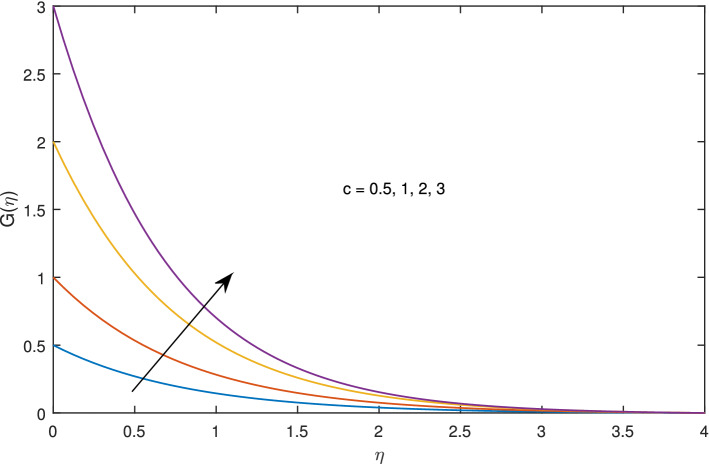
Effects of variation of *c* on azimuthal velocity, when $w_{\circ }=0.5$, $\zeta =0.1$, $\beta =0.2$, $N_{rd}=1$, $\phi =0.1$ and $Pr=6.2$.

**Figure 5:**
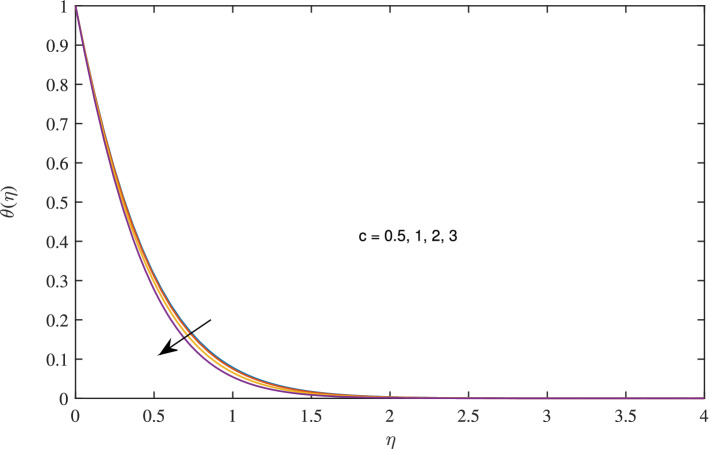
Effects of variation of *c* on temperature profile, when $w_{\circ }=0.5$, $\zeta =0.1$, $\beta =0.2$, $N_{rd}=1$, $\phi =0.1$ and $Pr=6.2$.

As the value of rotation parameter *c* boosts, the axial velocity *F* excites, as shown in Fig. 2. Physically, it is due to the centrifugal force that pumps the fluid in radial direction this vacancy is balanced by the fluid flowing in negative axial direction. As rotation parameter *c* is flourishing, radial velocity growing due to centrifugal force, as admitted in Fig. 3. It reflects from Fig. 4 that azimuthal velocity strengthen up as the values of rotational parameter *c* is advanced. Due to increase in values of *c* rotation velocity $\Omega$ get more strength. Figure 5 illustrates that as rotation parameter takes off the thermal boundary layer thickness is enervated which abates the temperature.

**Figure 6:**
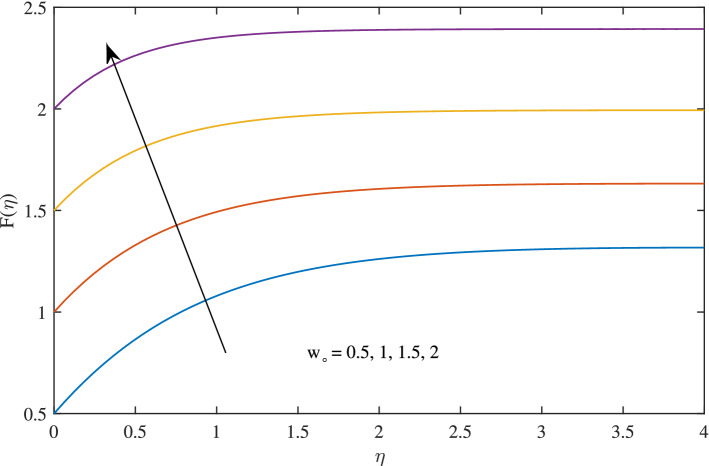
Effects of variation of suction parameter $w_{\circ }$ on axial velocity, when $c=1$, $\zeta =0.1$, $\beta =0.2$, $N_{rd}=0.2$, $\phi =0.1$ and $Pr=6.2$.

**Figure 7:**
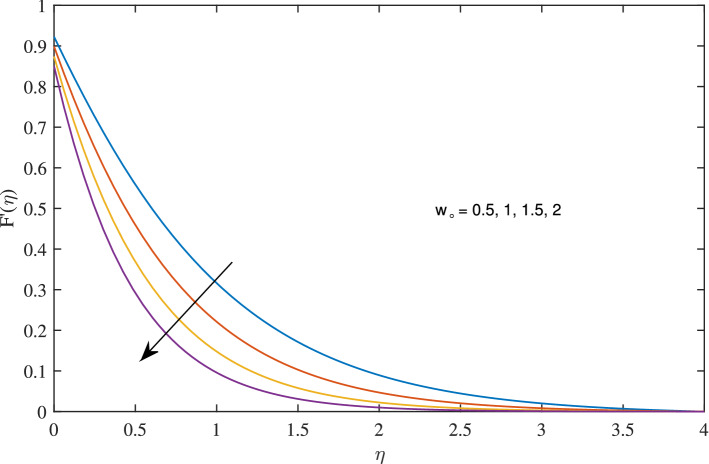
Effects of variation of suction parameter $w_{\circ }$ on radial velocity, when $c=1$, $\zeta =0.1$, $\beta =0.2$, $N_{rd}=0.2$, $\phi =0.1$ and $Pr=6.2$.

**Figure 8:**
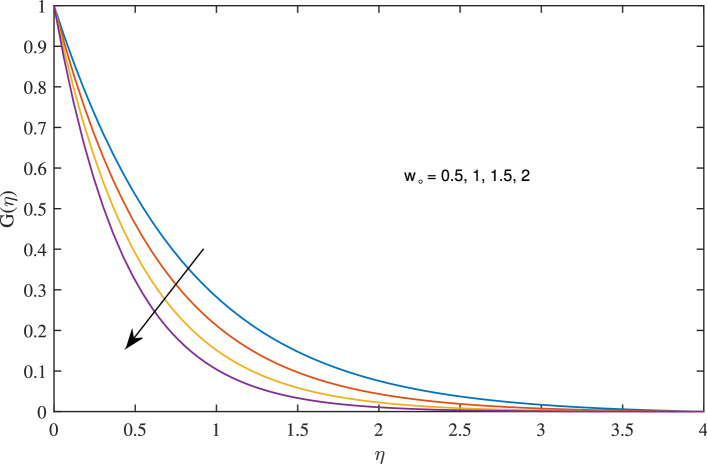
Effects of variation of suction on $w_{\circ }$ on azimuthal velocity, when $c=1$, $\zeta =0.1$, $\beta =0.2$, $N_{rd}=0.2$, $\phi =0.1$ and $Pr=6.2$.

**Figure 9:**
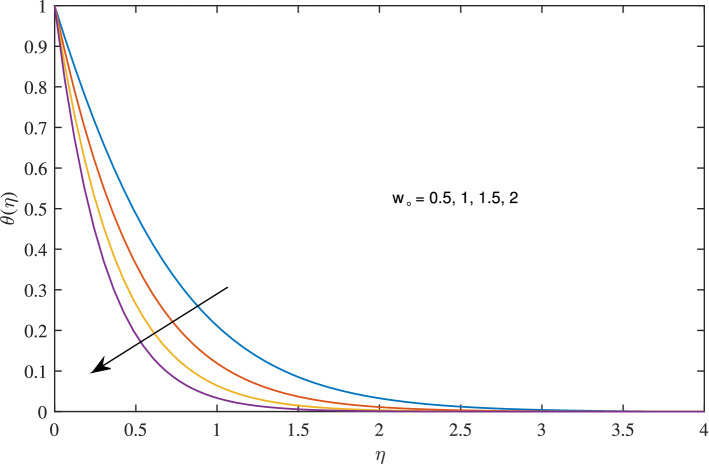
Effects of variation of suction parameter $w_{\circ }$ on temperature profile, when $c=1$, $\zeta =0.1$, $\beta =0.2$, $N_{rd}=0.2$, $\phi =0.1$ and $Pr=6.2$.

The consequences of variation of suction parameter $(w_{\circ }>0)$ on radial, axial, azimuthal velocities and temperature are translated in Figs. 6, 7, 8 and 9. When suction velocity jumps the axial velocity boosts, on other hand, deterioration in radial, azimuthal velocities and temperature is observed. It is due to the draw of fluid toward porous surface which responsible for decay in momentum boundary layer thickness .

**Figure 10:**
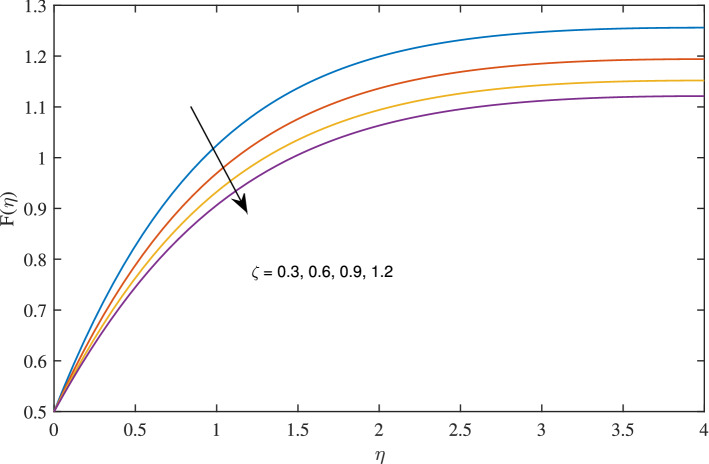
Effects of slip parameter $\zeta$ on axial velocity, when $c=1$, $w_{\circ }=0.5$, $\beta =0.2$, $N_{rd}=0.2$, $\phi =0.1$ and $Pr=6.2$.

**Figure 11:**
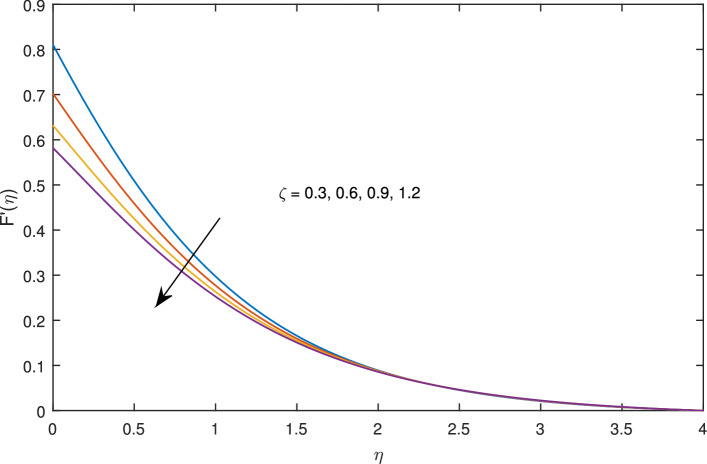
Effects of slip parameter $\zeta$ on radial velocity, when $c=1$, $w_{\circ }=0.5$, $\beta =0.2$, $N_{rd}=0.2$, $\phi =0.1$ and $Pr=6.2$.

**Figure 12:**
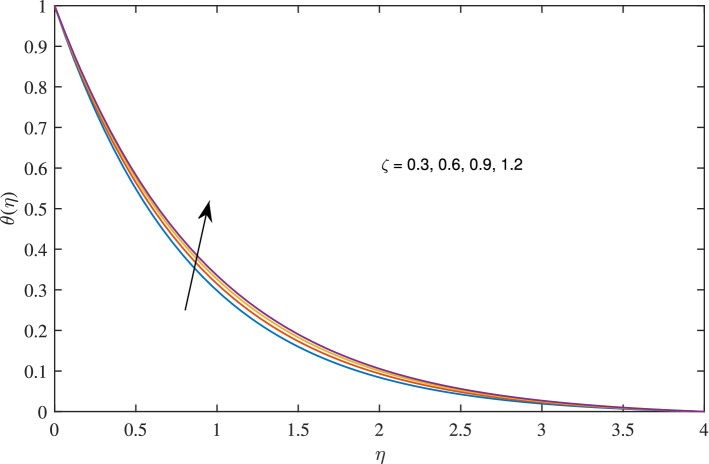
Effects of slip parameter $\zeta$ on azimuthal velocity, when $c=1$, $w_{\circ }=0.5$, $\beta =0.2$, $N_{rd}=0.2$, $\phi =0.1$ and $Pr=6.2$.

**Figure 13:**
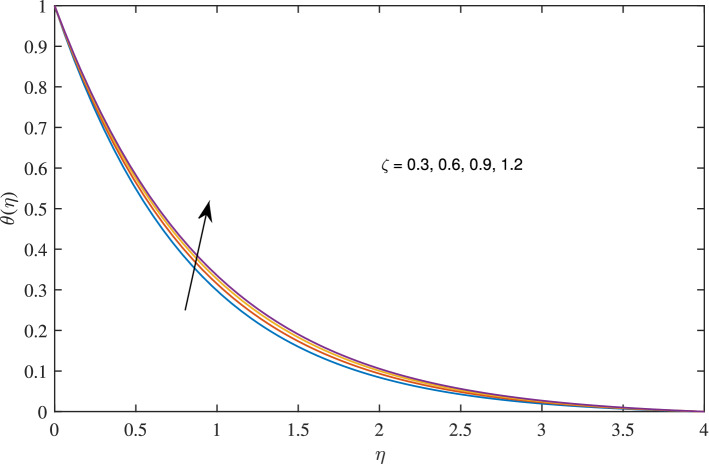
Effects of slip parameter $\zeta$ on temperature profile, when $c=1$, $w_{\circ }=0.5$, $\beta =0.2$, $N_{rd}=0.2$, $\phi =0.1$ and $Pr=6.2$.

In Figs. 10, 11, 12 and 13 insinuate the influence of Navier slip parameter $\zeta$ on velocity and temperature. It can be observed from Figs. 10 and 11 axial and radial velocities descend with development of the values $\zeta$, on the other hand, azimuthal velocity and thermal boundary layer increases with growth of $\zeta$, as shown in Figs. 12 and 13. Physically, friction is liable for such behavior of axial velocity *F*, radial velocity $F'$, azimuthal velocity *G* and temperature $\theta$ with the change in $\zeta$. Overall, increased wall slip causes to slip the fluid over the disk due to which significant fall in the coefficient of wall skin friction and Nusselt number is observed as reported in Tables 1 and 4. It is important to understand wall slippage for rheological analysis. As wall slippage is adverse in extrusion industry because it causes deformation and changes the manifestation of finishing products. The intensity of look distortion can be anywhere from a loss of shine or shark skin to complete melt crack. These appearance defects can notably affect production rate in built-up and therefore it is essential to comprehend the phenomena of wall slippage. Moreover, wall slip analysis is of vital importance for simulation and designing of injection molding process particularly micro-injection molding as wall slip is more noteworthy in small flow channels.

**Figure 14:**
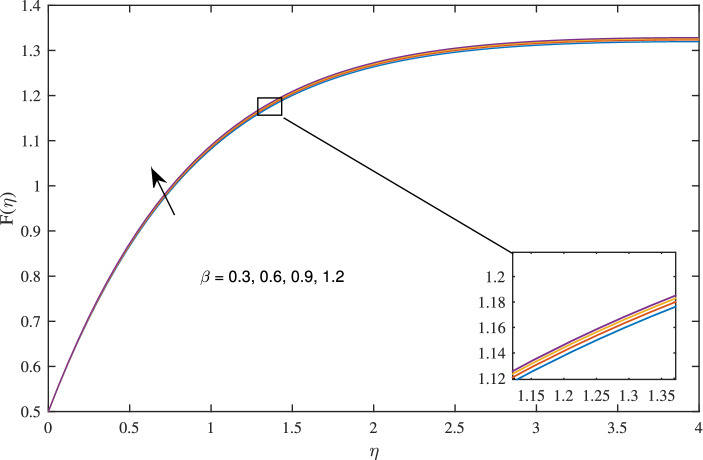
Effects of critical shear rate $$\beta $$ on axial velocity, when $$c=1$$, $$h=0.5$$, $$\zeta =0.1$$, $$N_{rd}=0.2$$, $$\phi =0.1$$ and $$Pr=6.2 $$.

**Figure 15:**
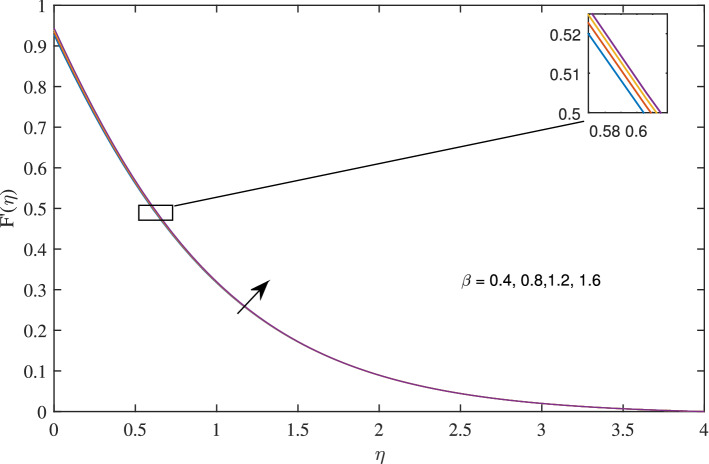
Effects of critical shear rate $$\beta $$ on radial velocity, when $$c=1$$, $$h=0.5$$, $$\zeta =0.1$$, $$N_{rd}=0.2$$, $$\phi =0.1$$ and $$Pr=6.2 $$.

**Figure 16:**
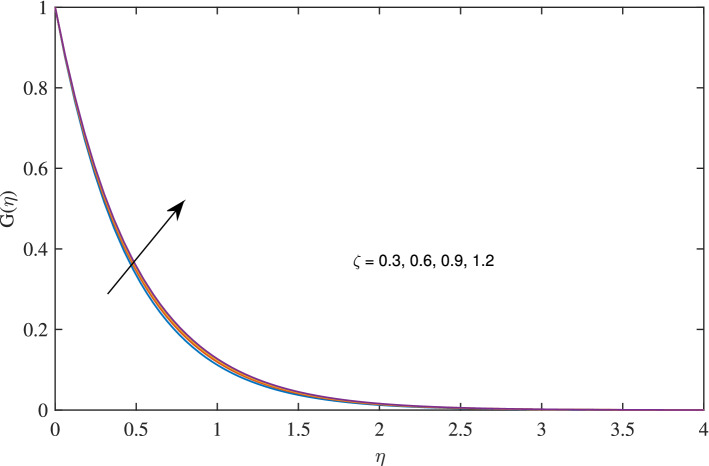
Effects of critical shear rate $$\beta $$ on azimuthal velocity, when $$c=1$$, $$h=0.5$$, $$\zeta =0.1$$, $$N_{rd}=0.2$$, $$\phi =0.1$$ and $$Pr=6.2 $$.

**Figure 17:**
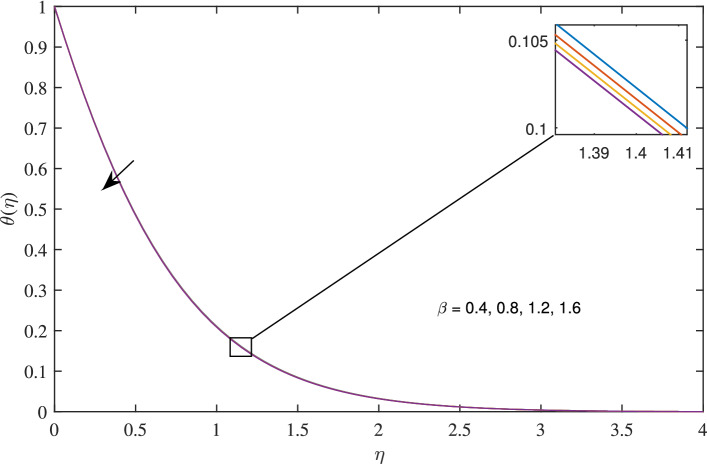
Effects of critical shear rate $$\beta $$ on temperature profile, when $$c=1$$, $$h=0.5$$, $$\zeta =0.1$$, $$N_{rd}=0.2$$, $$\phi =0.1$$ and $$Pr=6.2 $$.

Figures 14, 15, 16 and 17 elucidate that as reciprocal of critical shear rate $\beta$ prospers, the axial velocity *F*, radial velocity $F'$ become dominant and azimuthal velocity *G* and temperature $\theta$ reduced.

**Figure 18:**
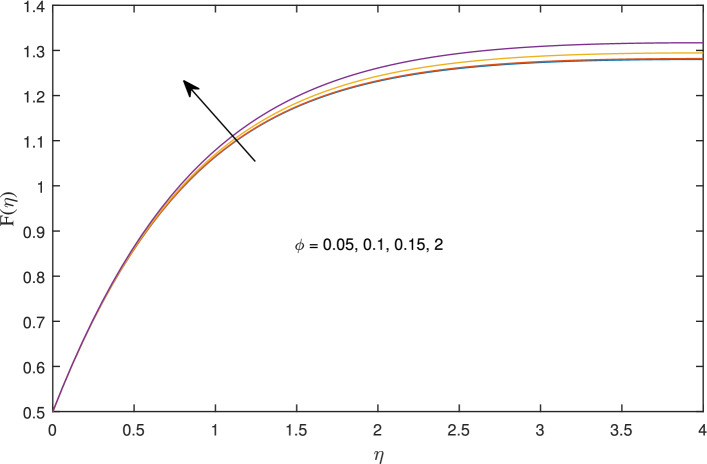
Effects of variation of nanoparticle volume fraction $\phi$ on axial velocity, when $c=1$, $w_{\circ }=0.5$, $\zeta =0.1$, $\beta =0.2$ $N_{rd}=0.2$, and $Pr=6.2$.

**Figure 19:**
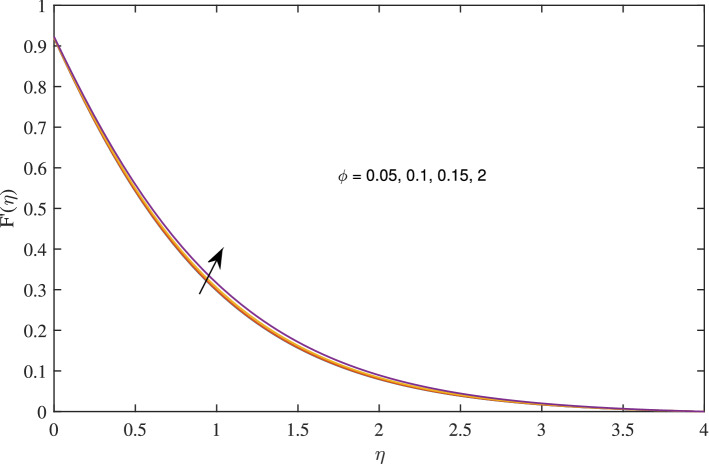
Effects of variation of nanoparticle volume fraction $\phi$ on radial velocity, when $c=1$, $w_{\circ }=0.5$, $\zeta =0.1$, $\beta =0.2$ $N_{rd}=0.2$, and $Pr=6.2$.

**Figure 20:**
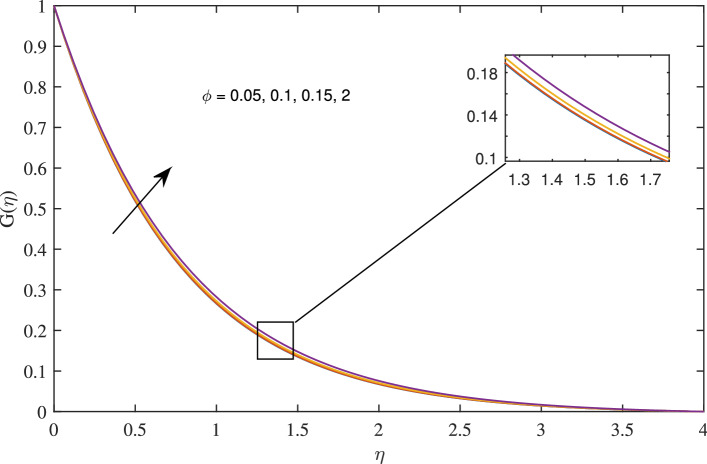
Effects of variation of nanoparticle volume fraction $\phi$ on azimuthal velocity, when $c=1$, $w_{\circ }=0.5$, $\zeta =0.1$, $\beta =0.2$ $N_{rd}=0.2$, and $Pr=6.2$.

**Figure 21:**
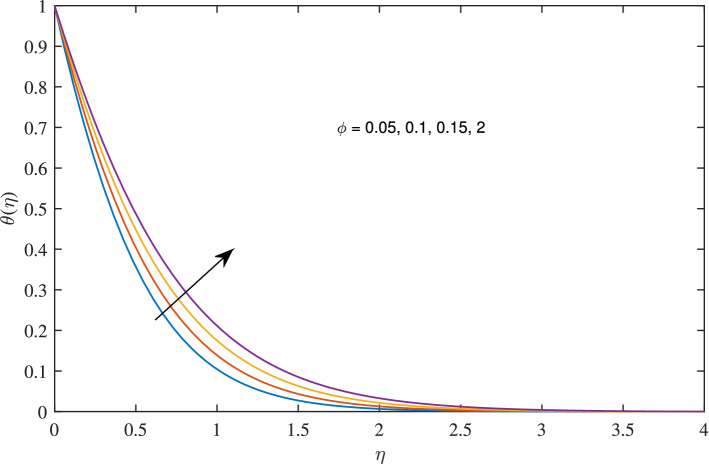
Effects of variation of nanoparticle volume fraction $\phi$ on temperature profile, when $c=1$, $w_{\circ }=0.5$, $\zeta =0.1$, $\beta =0.2$ $N_{rd}=0.2$, and $Pr=6.2$.

Figures 18, 19, 20 and 21 intimates that by the hike in nanoparticle fraction in base fluid the conduction of fluid amplifies, and conduction of $TiO_2$ over *Ag* and *Cu* dominate.

**Figure 22:**
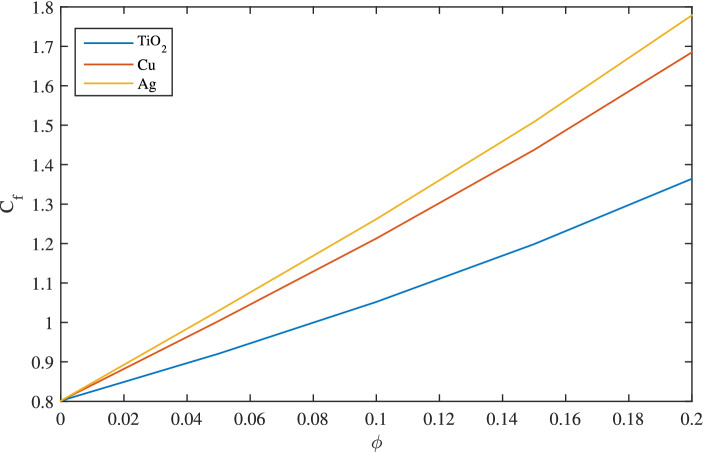
Graph of coefficient of skin friction for different nanoparticles, when $c=0.1$, $w_{\circ }=0.1$, $\zeta =0.1$,$\beta =0.2$ $N_{rd}=0.2$ and $Pr=6.2$.

**Figure 23:**
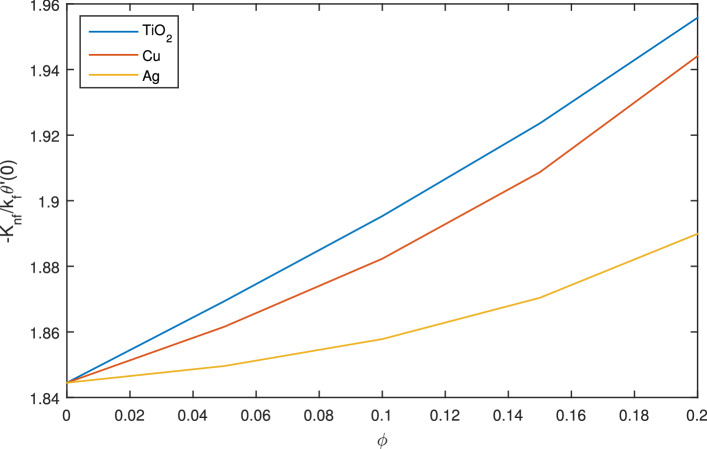
Graph of Nusselt number for different nanoparticles, when $c=0.1$, $w_{\circ }=0.1$, $\zeta =0.1$, $\beta =0.2$ $N_{rd}=0.2$ and $Pr=6.2$.

Figure 22 demonstrates the outcomes of skin friction coefficient $C_f$ for copper (*Cu*), sliver (*Ag*) and titanium dioxide $(TiO_2)$. Figure 22 indicates $TiO_2$ possesses less friction than *Ag* and *Cu*. In Fig. 23, Nusselts number is graphed against nanoparticle volume fraction $\phi$ of $TiO_2$, *Cu* and *Ag*. In case of $TiO_2$,high Nusselts number indicating high heat conduction as compared to *Cu* and *Ag*.

**Figure 24:**
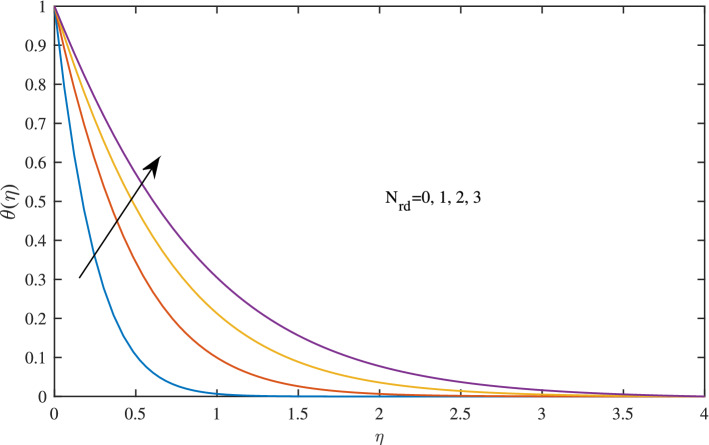
Effects of radiation parameter $N_{rd}$ on temperature profile, when $w{\circ }=0.5$, $\zeta =0.1$, $\beta =0.2$,$c=1$, $\phi =0.1$ and $Pr=6.2$.

Figure 24 demonstrates the influence of radiation parameter $N_{rd}$ on temperature profile $\theta (\eta )$. It is observed the temperature is increasing function of radiation parameter . The enhancement of radiative heat transfer is due to the fact of decreased mean absorption coefficient by increased value of radiation parameter $N_{rd}$ . It is noted that thermal boundary layer gets thick by varying radiation parameter $N_{rd}$.

## Conclusion

In this study, we examined the time independent flow and heat transfer of nanofluid over porous stretchable rotating disk observing nonlinear radiation and admitting slip in the presences of three types of nanoparticles: copper (*Cu*), silver (*Ag*), and titanium dioxide $(TiO_2)$. The governing equations of the problem are transformed into ordinary differential equations by Von Karman transformations and then solved by using bvp4c. The impacts on velocity and temperature profiles, of emerging quantities like, rotation parameter , nanoparticle concentration $\phi$, suction parameter $w_0$, slip parameters $\zeta$ , critical shear stress parameter $\beta$, and radiation parameter $N_rd$, are reported through several graphs and tables. To analyze the heat transfer process from wall to fluid the Nusselt number and skin friction coefficient are calculated and graphed against nanoparticles volume fractions. It is observed that titanium dioxide $(TiO_2)$ possesses less friction than silver (*Ag*) and copper (*Cu*), while high Nusselt number indicating high heat conduction as compared to copper (*Cu*) and silver(*Ag*). The consequences of variation of suction parameter $(w_0 > 0)$ on radial , axial, azimuthal velocities and temperature are also reported via several graphs and discovered that by enhancing the suction parameter, the axial velocity boosts, on other hand, deterioration in radial, azimuthal velocities and temperature is observed. Consequently which results in the decay of momentum boundary layer thickness . It is further noted that the increase in the radiation parameter results in the thickness of thermal boundary layer.

## Data Availability

The authors confirm that all data generated or analyzed that support the findings of the study are available within this article.
